# Comparative Analysis of Modulation Techniques on the Losses and Thermal Limits of Uninterruptible Power Supply Systems

**DOI:** 10.3390/mi13101708

**Published:** 2022-10-11

**Authors:** Edemar O. Prado, Pedro C. Bolsi, Hamiltom C. Sartori, José R. Pinheiro

**Affiliations:** 1 Energy Efficiency Lab, LABEFEA, Federal University of Bahia, Salvador 40170-110, BA, Brazil; 2 Power Electronics and Control Research Group, GEPOC , Federal University of Santa Maria, Santa Maria 97105-900, RS, Brazil

**Keywords:** electrical losses, modulation, power MOSFET, thermal analysis, uninterruptible power supply

## Abstract

This paper presents a comparative analysis of electrical losses and subsequent thermal limits of the inverter of UPSs for small office and home office (SOHO) applications. For this, three PWM modulation techniques applied to the full-bridge converter are considered, with power levels of 100–1000 W and switching frequencies of 30–120 kHz. To validate the electrical and thermal models, a dSapce MicroLabBox equipment was used to implement modulation techniques on a commercial 1000 W UPS, and a Keysight DAQ970A data logger was used for temperature measurements. As a result, the MOSFET temperatures and losses are obtained for the three modulation techniques evaluated, indicating the best scenario for use and its influence on the UPS autonomy time.

## 1. Introduction

Uninterruptible power supplies (UPSs) are electronic systems capable of supplying high-quality power to critical loads such as computers, data centers, medical and life support systems, communication systems, industrial controls, etc. [1,2,3,4,5,6]. In this type of application, UPSs are essential to maintain operation for certain periods of time, under normal or abnormal electrical power conditions, including interruptions from a few milliseconds to several hours [2,7].

UPS systems are classified into three types based on their operation and configuration. These three types include passive stand-by/offline, line-interactive, and double conversion/online [8,9,10,11]. Offline UPSs are the simplest configuration with only one power conversion stage. In the event of a grid failure, the batteries supply the load via an inverter. These types of UPS provide the least protection against grid voltage disturbances during normal operation, as the inverter operates only during backup mode [5,12,13].

Line-interactive UPSs are similar in configuration to offline UPSs; however, they include an AC line conditioning function to regulate the output and compensate grid undervoltages and overvoltages, providing a stable output voltage [10,11,12,13,14,15]. Both offline and line-interactive UPS use a transfer switch to disconnect the grid and connect the inverter when faults occur. Thus, the transfer time is on the order of a few milliseconds. [7,12,13].

In online UPSs, the AC input is converted to DC and then back to AC, keeping the amplitude and frequency stable at the load and providing a high degree of immunity to input voltage disturbances. In the event of an AC grid failure, the system immediately switches to backup mode [16,17]. With the AC–DC input stage, there is the possibility of the implementing power factor correction [16,17,18,19,20,21,22].

UPS systems cover a wide range of power levels, from single-phase systems rated at less than 1 kVA, to three-phase systems rated at more than 1000 kVA. High power systems, designed for large equipment and data centers, operate from battery banks with voltages in the range of hundreds of volts [16,17,21]. Smaller scale systems, for use in a small office or home office (SOHO) environments, generally include inverters that operate on 12 V or 24 V batteries and are mainly of offline and line-interactive type [16,17].

In SOHO UPSs, the battery bank is often connected to the load using a full-bridge converter and a low-frequency step-up transformer [16,17,23,24]. Due to the use of the transformer, the inverter current is higher than the load current, resulting in a high current stress on the inverter transistors [24]. Depending on the power of the converter, MOSFETs are be used in parallel to withstand higher currents [16,17].

To obtain reduced ripples of voltage and current and lower total harmonic distortion (THD) in the output voltage, three-level pulse-width modulations are applied to the inverter [25,26,27,28]. Among the three-level PWM modulation techniques, three are commonly applied to the full-bridge converter [25,28]: the discontinuous modulation, where each converter leg operates at high frequency in one half-cycle and remains either on or off in the next half-cycle; the phase-shifted modulation, with both legs operating at high frequency; and the discontinuous single-phase leg switched, where one leg of the converter operates at high frequency and the other leg operates at the grid frequency.

Considering the aforementioned modulation techniques, this work evaluates their performance on full-bridge inverters applied to SOHO UPSs. The analysis is made for backup mode operation, with a rated battery voltage of 24 V, rated power of 1 kW, and RMS output voltage of 120 V (60 Hz). To reduce the current on the transistors, two MOSFETs are used in parallel. The main contribution of this work is the comparative analysis of electrical losses and thermal limitation in the inverter for: different modulation techniques applied to the full-bridge converter, two different turn ratios in the transformer, power variations in the load (100–1000 W), and switching frequencies of 30–120 kHz.

This paper is organized as follows. Section 2 describes the small office and home office UPS application. Section 3 describes the modulation techniques used in the comparative analysis. Section 4 shows the experimental validation of the computational and thermal models. Section 5 presents the results obtained for the operation points detailed above, and Section 6 concludes the paper.

## 2. UPSs for Applications of Small Office and Home Office (SOHO)

SOHO UPSs are mainly of offline and line-interactive type, with square or sine wave output waveforms, being the latter more common [16,17]. The inverter for SOHO UPSs generally operates from a battery voltage of 12 V or 24 V [17], making it necessary to raise the voltage at the output of the inverter using a low-frequency transformer [16,17,23,24]. Topologies that employ a low-frequency transformer are known as ferroresonant-based UPSs [29,30,31,32,33].

The battery bank is connected to the primary winding of the step-up transformer through a push-pull or full-bridge converter. Push-pull and full-bridge inverter topologies are widely used in UPS systems, as they utilize transformer efficiency to maximize power transfer capacity for available volume [17,34]. An advantage of the full-bridge converter over the push-pull topology is that the primary winding of the transformer works in both polarities without the need for a center tap, which allows for a reduction in volume [16,17] .

In full-bridge systems with a sinusoidal output waveform, the transformer is driven by a high-frequency PWM switching voltage, and the output is filtered so that

(1) $$V_{OUT}\left(t\right)=\left(\pm \right)\frac{N_{S}}{N_{P}}m_{a}\left(t\right)V_{IN}$$

where $V_{IN}$ is the battery bank voltage and $\frac{N_{S}}{N_{P}}$ is the turn ratio of the transformer. A time-varying modulated duty cycle function $m_{a}\left(t\right)$ is used to adjust the output voltage $V_{OUT}\left(t\right)$. The high leakage inductance inherent to low-frequency transformers is combined with an output capacitor to create a low-pass filter, attenuating the THD at the output [16,17,29].

**Figure 1:**
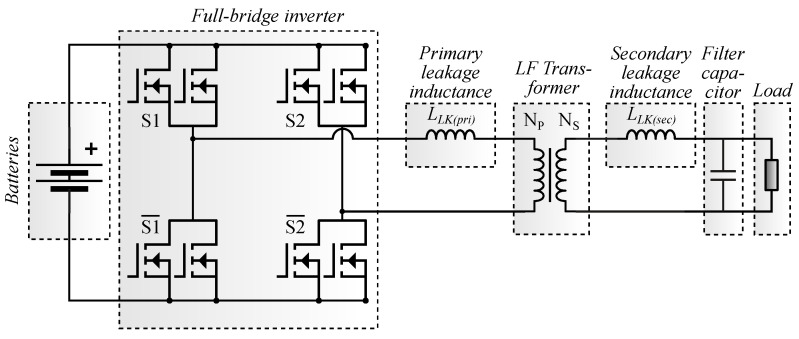
Offline or line-interactive ferroresonant-based UPS operating in backup mode.

The maximum drain–source voltage on the MOSFETs is equal to the voltage drop across their intrinsic diode plus the battery voltage; in addition to a small overshoot in the turn-off transient. Therefore, the MOSFET blocking voltage rating ($V_{DSB}$) can be selected based on the highest battery voltage plus a safety margin. The electrical circuit of a full-bridge converter-based UPS operating in backup mode, with two MOSFETs in parallel, is shown in Figure 1. This configuration is employed by both offline and line-interactive ferroresonant-based UPSs.

## 3. Three-Level PWM Applied to Single-Phase Full-Bridge Inverter

**Figure 2:**
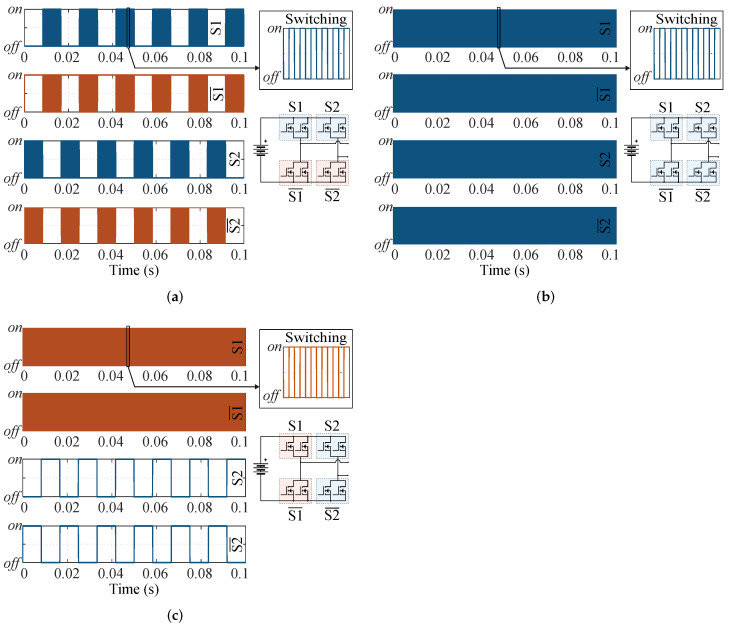
Three-level modulations were applied to a single-phase full-bridge inverter. (**a**) MT1. (**b**) MT2. (**c**) MT3.

Three three-level PWM techniques are applied to a full-bridge converter to comparatively analyze electrical losses, temperature, and harmonic content. In Figure 2, six cycles of the activation signals are shown, identifying each modulation technique as MT1, MT2, and MT3, defined as:

- MT1: discontinuous modulation, where only one phase leg is modulated at high frequency in the first half of the fundamental cycle, while the other phase leg is modulated in the second half of the fundamental cycle. The phase legs never switch over within the same carrier period [25].
- MT2: phase-shifted, where both legs of the converter operate at high-frequency [25].
- MT3: discontinuous single-phase leg switched, where one leg works at high frequency and the other at the fundamental cycle frequency [25].

In MT1 (Figure 2a), the switches in blue color represent transistors S1 and S2. The placement of these transistors can be verified in the schematic of the inverter. The orange color represents the complementary transistors $\bar{S1}$ and $\bar{S2}$. To attain a frequency at the output of the converter ($F_{OUT}$) of 30 kHz, the switching frequency ($F_{SW}$) of the transistors is 30 kHz. The transistors S1 and S2 operate a half-cycle at the switching frequency and another half-cycle in off-state. $\bar{S1}$ and $\bar{S2}$ switch in one half-cycle and in the other half-cycle remain on.

In MT2 (Figure 2b), the modulation strategy presents symmetrical switching for the four transistor positions (S1, $\bar{S1}$, S2, and $\bar{S2}$). In this modulation, there is only one switching pattern. To attain $F_{OUT}\;=$ 30 kHz, the $F_{SW}$ of the transistors is one-half of $F_{OUT}$, i.e., 15 kHz.

In MT3 (Figure 2c), the switches in orange represent transistors S1 and $\bar{S1}$, and the blue color identifies transistors S2 and $\bar{S2}$. For an $F_{OUT}$ of 30 kHz, the $F_{SW}$ of transistors S1 and $\bar{S1}$ is 30 kHz, S2 and $\bar{S2}$ operate at 60 Hz.

## 4. Experimental Validation of Computational and Thermal Models

### 4.1. Computational Models and UPS Waveforms

**Figure 3:**
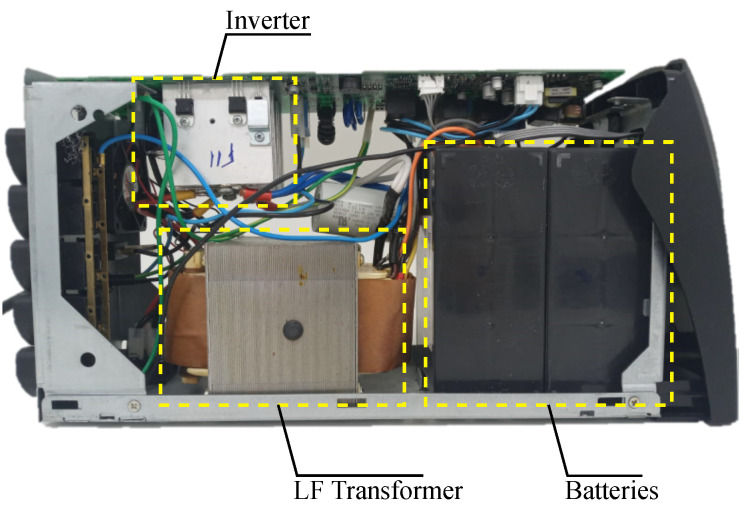
Commercial line-interactive ferroresonant-based UPS of 1 kW.

The methodology of analysis is applied to a commercial line-interactive UPS of 1 kW shown in Figure 3. The backup mode equivalent circuit is shown in Figure 1. The RMS voltage at the output is 120 V (60 Hz), and the power supply consists of a pair of 12 V/7 Ah batteries connected in series (24 V), with support for an external parallel battery bank to increase the current capacity (Ah). The part number of the silicon MOSFET under analysis is STP220N6F7 [35].

**Figure 4:**
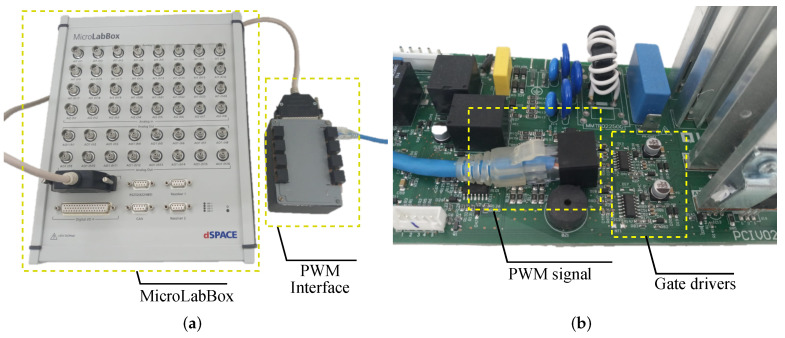
Connections for modifying UPS modulation. (**a**) dSpace MicroLabBox equipment. (**b**) Connection of signals from dSpace via RJ45.

The modulation techniques presented in Section 3 were implemented using a dSpace MicroLabBox equipment. To isolate the gate drivers and MOSFETs from the remaining hardware of the commercial UPS, its microcontroller and control circuits were disconnected, and replaced by dSpace MicroLabBox. This equipment has communication with MATLAB Simulink^®^ software and sends the PWM signals via RJ45 connection to the gate drivers. The use of this equipment allows for changes in the switching frequency in real time, through an interface in the controldesk software. The dSpace MicroLabBox equipment and the PWM interface are shown in Figure 4a, and the connection of signals from dSpace via RJ45 can be seen in Figure 4b.

**Figure 5:**
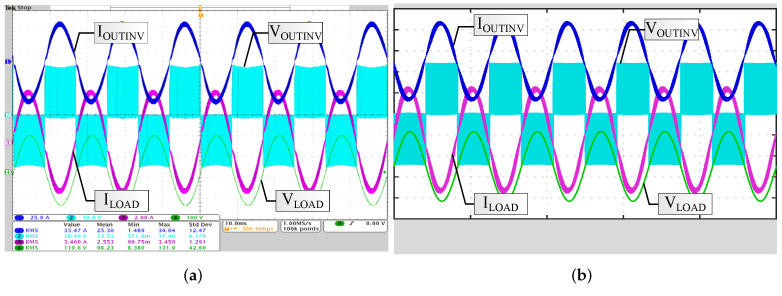
Waveforms with 400 W load. (**a**) Experimental. (**b**) Simulation.

In MATLAB Simulink^®^ the UPS is simulated, including inverter topology, modulation, and transformer impedance. Voltage drops in batteries, cables, and connectors are also modeled. In this way, the RMS values and THDs of the current and voltage waveforms reproduce the experimental values. Figure 5a presents the measured waveforms of voltage and current at the inverter output and at a 400 W load, and Figure 5b the simulated waveforms. In this example, MT1 is used along with a transformer turns ratio of $N_{S}/N_{P}=9.5$. The RMS values of voltage and current at the inverter output and at the load are shown in Table 1, comparing the simulated values to those obtained experimentally (using dSpace).

### 4.2. Model and Methodology for Losses Calculation

For the calculation of conduction ($P_{COND}$), switching ($P_{SW}$) and reverse recovery ($P_{rr}$) losses, the models presented in [36,37] are used. The conduction losses are evaluated as a function of the junction temperature. For switching losses, the Miller capacitance is evaluated as a function of the drain–source voltage over the MOSFET.

**Figure 6:**
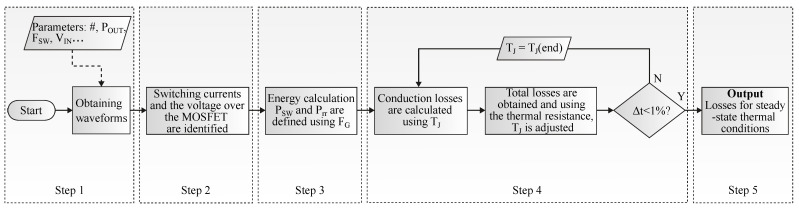
Flowchart for calculating losses in power MOSFETs.

To calculate losses at different operation points of frequency and power levels, an iterative algorithm between MATLAB script^®^ and MATLAB Simulink^®^ circuit simulation is employed. Figure 6 shows the flowchart for calculating losses in each transistor of the full-bridge inverter. Its steps are numbered from 1 to 5:

- Step 1: system inputs are defined: part number of the MOSFET, power levels, switching frequency range, and input voltage. These data are loaded into MATLAB Simulink^®^ and the waveforms are obtained;
- Step 2: the switching currents and the voltage over the MOSFET are identified;
- Step 3: the energies related to the turn-on and turn-off for each switching cycle are calculated, and using the grid frequency ($F_{G}$), $P_{SW}$ and $P_{rr}$ are defined;
- Step 4: the calculation of conduction losses is performed using the junction temperature ($T_{J}$) and the drain–source on-state resistance ($R_{DSon}$) given in the datasheet. Total losses are obtained by adding $P_{SW}$, $P_{rr}$, and $P_{COND}$. Based on the junction-case and case-ambient thermal resistances, $T_{J}$ is calculated. As $T_{J}$ influences $R_{DSon}$, and the increase in $R_{DSon}$ increases the losses, and $T_{J}$ [38], the loss calculation process is repeated. This process runs until the difference between the $T_{J}$ iterations is <1% (MOSFET thermal steady-state operating point).
- Step 5: as output, the losses for thermal steady-state conditions are defined.

### 4.3. Thermal Models

As mentioned in [36,37,38,39,40], the $R_{DSon}$ of the MOSFET is a function of $T_{J}$. Thus, it is necessary to obtain thermal models that represent the temperature variation over time. For this, the thermal behavior of the MOSFETs was measured using a Keysight DAQ970A data logger.

**Figure 7:**
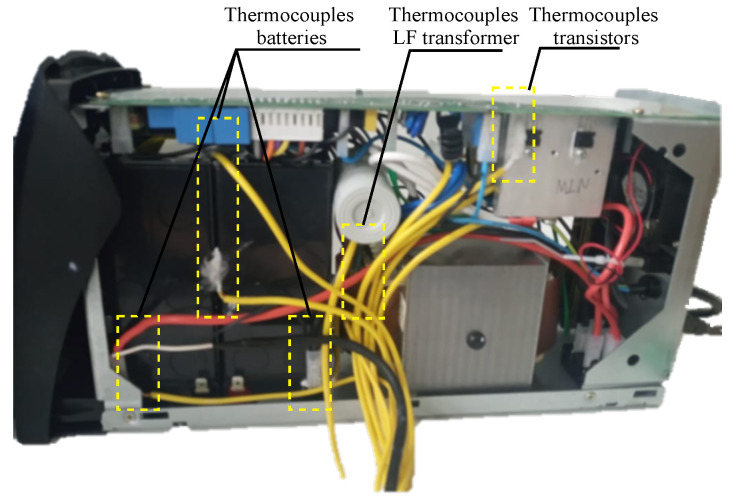
Connection of thermocouples in the UPS.

In Figure 7, the connections of the thermocouples in the UPS are shown. These were placed to obtain the temperatures in the transistors of each pair and also added to different parts of the circuit for thermal monitoring. The thermocouples used are of the K-type, which have an accuracy of ±2%.

**Figure 8:**
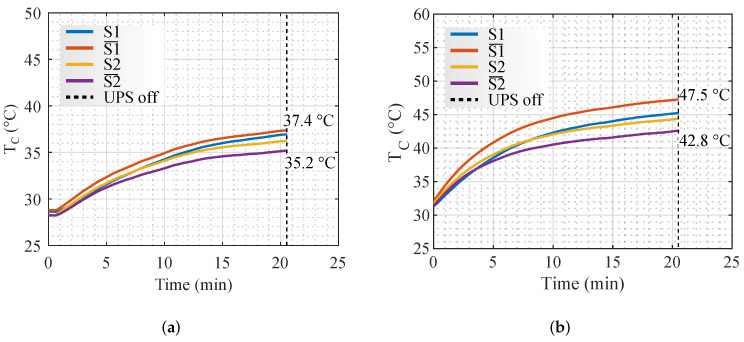
Temperatures obtained experimentally in the MOSFETs. (**a**) 400 W. (**b**) 600 W.

With the default battery bank of 7 Ah batteries, the temperature in the MOSFETs does not reach the steady-state condition before the batteries are completely discharged. To increase the autonomy of the UPS and emulate the external battery bank, a voltage source model Itech IT7900 was used. The resulting MOSFET case temperatures ($T_{C}$) are shown in Figure 8a,b for the operating points of 400 W and 600 W, respectively.

The behavior of temperature as a function of time can be represented in a simplified way by a parallel RC circuit (equivalent thermal resistance and capacitance) [41,42,43,44,45]. To determine the equivalent thermal resistance, the temperature obtained in thermal steady-state is used,

(2) $$T\left(t\right)=PR_{th}+T_{A}.$$

The equivalent thermal capacitance that models the behavior of temperature over time is obtained by extracting a point from the thermal transient,

(3) $$T\left(t\right)=PR_{th}\left(1-e^{\frac{-t}{R_{th}C_{th}}}\right)+T_{A}.$$

To obtain the coefficients of (3), the temperature at five minutes was used. In Table 2, the resistances and thermal capacitances obtained from the curves of Figure 8 are shown.

Due to the small divergence among the coefficients in the different evaluated powers, the average between them was used. It is worth mentioning that these coefficients are only valid for the UPS under study. In case other hardware is used, or if there are layout modifications (such as repositioning the air cooler or changing the heatsink), these coefficients will change and must be recalculated following the same methodology.

Based on the results of Table 2, for the numerical analysis of the following sections, the $R_{TH}$ and $C_{TH}$ used for all transistors are 8.4 °C/W and 1 J/°C (average among S1, $\bar{S1}$, S2 and $\bar{S2}$). In this way, only the effect of each modulation on temperature is evaluated, disregarding issues related to the heat transfer system.

## 5. Influence of Modulation Techniques on Inverter Losses and Temperature

### 5.1. Loss Comparison

Losses were obtained for MT1, MT2, and MT3 in two transformer turn ratio scenarios. These scenarios were considered because the turns ratio influences the current and the modulated duty cycle (1), according to the voltage in the inverter output. In the first scenario, the losses and temperatures are analyzed considering $N_{S}/N_{P}=9.5$. In the second scenario, $N_{S}/N_{P}=11.9$. The inverter configuration uses two MOSFETs in parallel in each heatsink, as shown in Figure 1 and Figure 3.

#### 5.1.1. Scenario 1: Transformer Turn Ratio of $N_{S}/N_{P}=9.5$

Table 3 shows the calculated losses for power levels of 100 W to 1000 W with *steps* of 100 W, $F_{OUT}$ = 30 kHz, and thermal steady-state conditions. The values presented in Table 3 correspond to the sum of losses of the two MOSFETs in parallel. Table 4 shows the total losses in the inverter for each modulation technique.

In the total losses shown in Table 4, a negligible difference in losses using MT1, MT2, and MT3 was verified. The biggest difference among the modulations is in the individual losses per position of MOSFETs, as shown in Table 3. Considering the worst-case losses (1000 W load) in MT1, losses are 3.9 W in S1 and S2 and 8.1 W for the complementary transistors $\bar{S1}$ and $\bar{S2}$. This is because of the asymmetry of the modulation, which concentrates most of the current in the complementary transistors. In MT2, the losses are 6.34 W in all transistors. In MT3, the transistors S1 and $\bar{S1}$ concentrate the switching losses and present the highest loss, being 7.3 W, while S2 and $\bar{S2}$ operate at grid frequency (60 Hz) with losses of 5.5 W.

**Figure 9:**
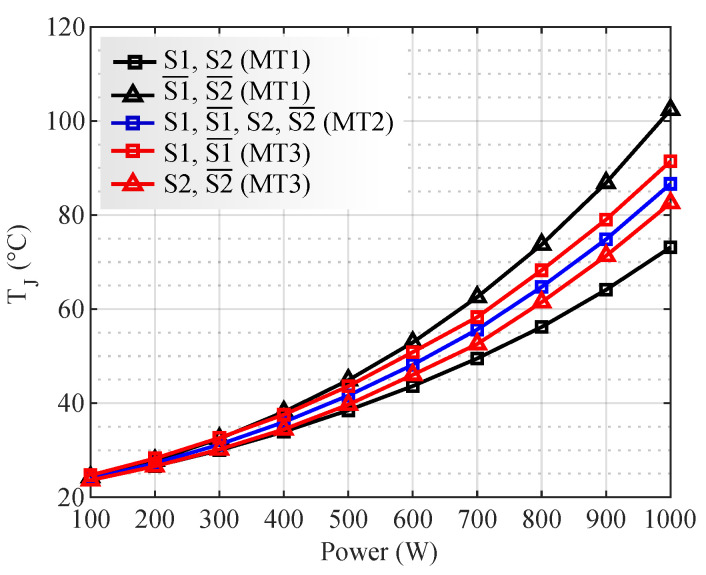
Transistor junction temperatures for each modulation technique, $N_{S}/N_{P}$ = 9.5.

The junction temperatures are shown in Figure 9. MT1 is represented by black lines (squares for S1 and S2 and triangles for $\bar{S1}$ and $\bar{S2}$). MT2, which has loss symmetry, is represented by a single blue line. The red lines represent MT3 (squares for S1 and $\bar{S1}$ and triangles for S2 and $\bar{S2}$.

In Figure 9, for MT1, it can be seen that transistors $\bar{S1}$ and $\bar{S2}$ present higher temperatures than S1 and S2. This thermal imbalance comes from the asymmetry of the modulation, which concentrates most of the current in $\bar{S1}$ and $\bar{S2}$. MT3 also presents a thermal imbalance, as it concentrates the switching losses in transistors S1 and $\bar{S1}$. MT2 presents intermediate temperatures relative to the other modulations, with thermal symmetry among transistors.

#### 5.1.2. Scenario 2: Transformer Turn Ratio of $N_{S}/N_{P}=11.9$

Table 5 shows a comparison among the total losses in the inverter connected to a transformer with $N_{S}/N_{P}=9.5$ (at the top of the table) and $N_{S}/N_{P}=11.9$ (highlighted in bold at the bottom of the table), for 800 W, 900 W and 1000 W. The increase in losses associated with the change in transformer turns ratio was similar for the three modulations evaluated, representing increments of 55.5% at 800 W, 56.5% at 900 W, and 56.8% at 1000 W.

**Figure 10:**
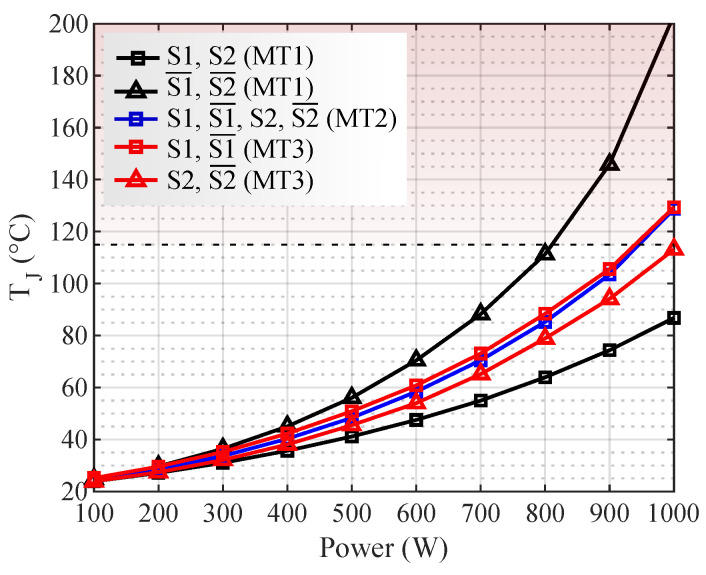
Transistor junction temperatures for each modulation technique, $N_{S}/N_{P}$ = 11.9.

The junction temperatures on the transistors for each modulation are shown in Figure 10. In it, the horizontal dashed line represents a temperature limit for the junction temperature, set at 115 °C in this work to ensure the safe operation of the MOSFETs. The maximum junction temperature provided by the manufacturer is 175 °C.

**Figure 11:**
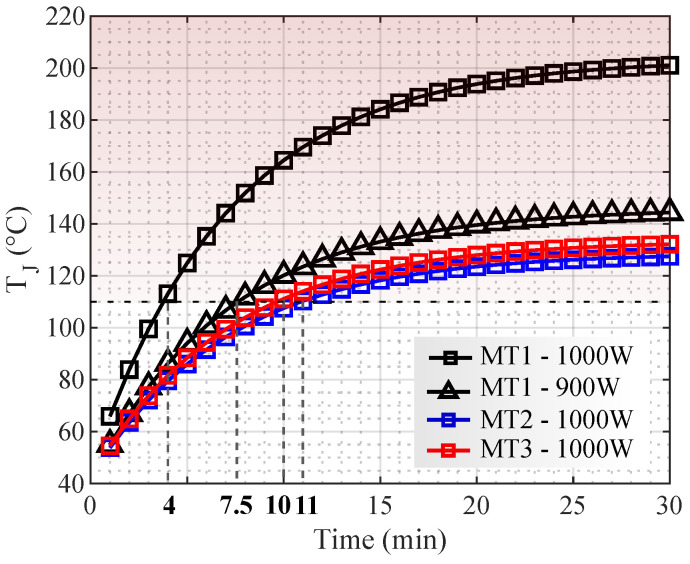
Thermal transient with transformer turn ratio of $N_{P}/N_{S}$ = 11.9.

In Figure 10, for powers higher than 810 W, the transistors $\bar{S1}$ and $\bar{S2}$ exceed the established thermal limit with MT1. In MT2 and MT3, there are limitations with powers higher than 950 W. During UPS operation, if the temperature limit is exceeded, the system shuts down due to transistor overheating. Thus, the temperature steady-state is not reached before the UPS shuts down in these cases. Figure 11 shows the thermal transient for the load conditions where the maximum temperature was exceeded. For MT1, the converter operation time was 9 min for 900 W and 4 min for 1000 W. In MT2, the converter operation was limited to 13 min with 1000 W. In MT3, the time limit was 11.5 min with 1000 W.

### 5.2. Thermal Analysis of The Influence of Frequency Variation

**Figure 12:**
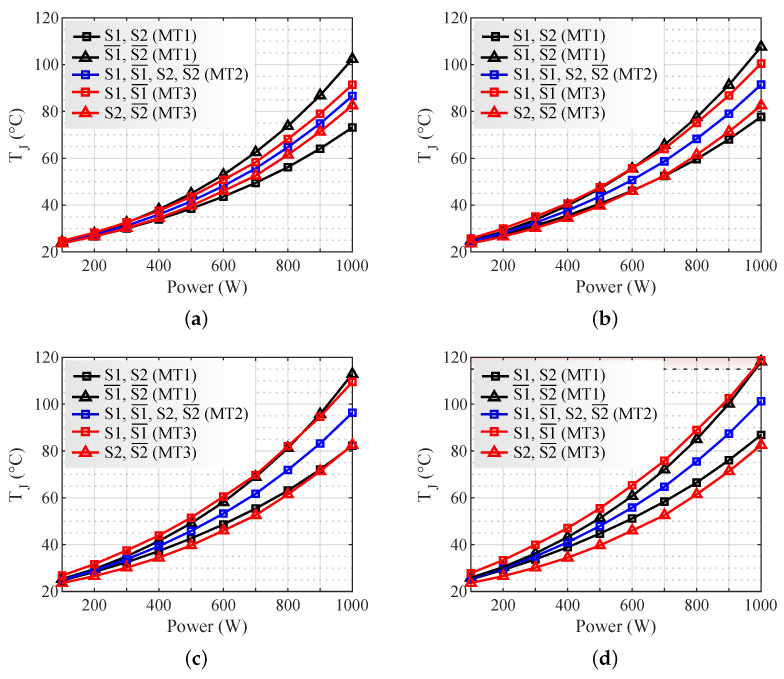
Junction temperatures for $N_{S}/N_{P}=9.5$ and various $F_{OUT}$ (**a**) 30 kHz. (**b**) 60 kHz. (**c**) 90 kHz. (**d**) 120 kHz.

To analyze the impact of frequency variation, the frequencies at inverter output $F_{OUT}$ = 30 kHz, 60 kHz, 90 kHz, and 120 kHz were considered, with a turn ratio of $N_{S}/N_{P}=9.5$. In Figure 12 the junction temperatures are shown as a function of $F_{OUT}$ for the different evaluated modulation techniques, being the $F_{OUT}$ of Figure 12 (a) 30 kHz, (b) 60 kHz, (c) 90 kHz, and (d) 120 kHz.

At 30 kHz (Figure 12a) MOSFETs $\bar{S1}$ and $\bar{S2}$ with MT1 present the highest junction temperatures (102 °C at 1000 W). In MT2, all transistors present the maximum temperatures of 86 °C. In MT3, S1 and $\bar{S1}$ presented maximum temperatures of 91 °C. As $F_{OUT}$ increases, the thermal imbalance in MT3 increases. This is because MT3 concentrates on switching losses only on transistors S1 and $\bar{S1}$.

For 120 kHz (Figure 12d), at 1000 W, the maximum and minimum temperatures in MT1 are 120 °C and 85 °C; in MT2, the thermal equilibrium of the transistors is at 101 °C; and the maximum and minimum temperatures of MT3 are at 120 °C and 81 °C. The temperature exceeds the established thermal limit of 115 °C at the transistor junction for MT1 and MT3 at 1000 W. In this condition, the autonomy was reduced to approximately 22 min in both. In MT2, there were no thermal limitations.

Overall, MT1 presents asymmetry in the conduction losses. Therefore, it tends to imbalance as the power increases. To use this modulation strategy, it is necessary to compensate for thermal asymmetry, reducing the $R_{th}$ of the transistors that are kept on during a half-cycle ($\bar{S1}$ and $\bar{S2}$). MT2 has symmetry in the conduction and switching losses. Its use is recommended in symmetrical heat transfer system ($R_{th}$) applications. MT3 presents asymmetry in switching losses, thus, it increases the thermal imbalance as the $F_{SW}$ rises. To use this modulation strategy, it is necessary to compensate for thermal asymmetry by reducing the $R_{th}$ of the leg that works at high $F_{SW}$.

## 6. Conclusions

In this paper, a comparative analysis among three PWM techniques was performed, evaluating the electrical losses in the inverter and the thermal limitation points of a 1000 W line-interactive UPS. The modulation techniques were applied to a full-bridge converter, considering discontinuous, phase-shifted, and discontinuous single-phase leg switched PWM, operating with different transformer turns ratios, load powers of 100–1000 W, and frequencies of 30–120 kHz.

In relation to total losses, the three evaluated modulation techniques presented similar results. When analyzing the losses for each MOSFET position individually, the impact of each modulation technique can be seen. MT1 concentrates most of the currents in the complementary transistors ($\bar{S1}$ and $\bar{S2}$), which increases their losses. MT2 distributes the losses evenly in all transistors. In MT3, the the switching losses are concentrated mostly in S1 and $\bar{S1}$, thus, the losses of the leg that operates at high frequency (S1 and $\bar{S1}$) are greater than the one that operates at low frequency (S2 and $\bar{S2}$).

In the comparative analysis, it was shown how the losses resulting from each modulation technique directly influence thermal behavior. Where there is an imbalance of losses, there is a thermal imbalance among the transistors. For the transformer turn ratio of $N_{S}/N_{P}=9.5$, working with an output high frequency harmonic of 30 kHz, the temperatures for all modulations remained in the safe operating region, with a maximum temperature of 102 °C in the transistors $\bar{S1}$ and $\bar{S2}$ of MT1. For the transformer turn ratio of $N_{S}/N_{P}=11.9$, the increase in current increases the transistor losses and temperatures. With this turn ratio and 30 kHz, for MT1, the specified thermal limit for the MOSFETs was reached after 9 min for 900 W and 4 min for 1000 W. In MT2, the converter operation was limited to 13 min and in MT3 to 11.5 min, both for 1000 W.

The frequencies evaluated were 30 kHz, 60 kHz, 90 kHz, and 120 kHz. In MT1, asymmetry was present in all frequencies, being the transistors that operate a half-cycle always on ($\bar{S1}$ and $\bar{S2}$) those with the highest temperatures. Since in MT3 switching losses are concentrated mostly on transistors S1 and $\bar{S1}$, the thermal imbalance increases as the frequencies increase. For the power of 1000 W and 120 kHz, the temperature exceeds the established thermal limit of 115 °C at the transistor junction for MT1 and MT3. In this condition, the autonomy was limited to approximately 22 min in both. In MT2, there were no thermal limitations.

## Figures and Tables

**Table 1:** Experimental and simulation voltage and current RMS values, with a load of 400 W.

	Experimental	Simulation
**Current at the inverter output**	33.47 A	33 A
**Voltage at the inverter output**	16.49 V	16.44 V
**Current at the load**	3.4 A	3.38 A
**Voltage at the load**	119.6 V	120 V

**Table 2:** Thermal coefficients calculated.

	$R_{\mathrm{TH}}$ (°C/W)			$C_{\mathrm{TH}}$ (J/°C)		
	**400 W**	**600 W**	**Average**	**400 W**	**600 W**	**Average**
**S1**	10.5	10.5	10.5	0.85	0.85	0.85
** $\bar{S1}$ **	7.2	7.2	7.2	1.1	1.1	1.1
**S2**	9.7	10.2	10	0.8	0.75	0.77
** $\bar{S2}$ **	5.6	5.8	5.7	1.4	1.3	1.35

**Table 3:** MOSFET Losses: $R_{TH}$ = 8.4 °C/W, part number STP220N6F7, thermal steady-state condition.

MOSFET Losses (W)												
	**MT1**				**MT2**				**MT3**			
**Power (W)**	**S1**	$\bar{S1}$	**S2**	$\bar{S2}$	**S1**	$\bar{S1}$	**S2**	$\bar{S2}$	**S1**	$\bar{S1}$	**S2**	$\bar{S2}$
100	0.09	0.15	0.09	0.15	0.17	0.17	0.17	0.17	0.30	0.31	0.09	0.08
200	0.21	0.37	0.21	0.37	0.38	0.37	0.38	0.37	0.57	0.58	0.23	0.23
300	0.39	0.72	0.39	0.72	0.68	0.67	0.69	0.68	0.95	0.95	0.45	0.46
400	0.63	1.20	0.63	1.19	1.08	1.08	1.08	1.08	1.44	1.41	0.77	0.77
500	0.94	1.81	0.94	1.81	1.58	1.59	1.58	1.58	2.03	2.02	1.19	1.20
600	1.32	2.59	1.33	2.59	2.20	2.20	2.20	2.20	2.74	2.69	1.74	1.75
700	1.80	3.59	1.79	3.59	2.95	2.95	2.96	2.95	3.66	3.57	2.41	2.38
800	2.36	4.79	2.36	4.82	3.90	3.89	3.90	3.89	4.68	4.52	3.27	3.23
900	3.07	6.28	3.07	6.28	5.00	5.00	5.00	5.00	5.97	5.78	4.30	4.22
1000	3.90	8.11	3.90	8.11	6.34	6.34	6.34	6.33	7.36	7.39	5.42	5.56

**Table 4:** Total inverter losses: $R_{TH}$ = 8.4 °C/W, part number STP220N6F7, thermal steady-state condition.

$F_{\mathrm{OUT}}$ = 30 kHz			
**Power (W)**	**MT1**	**MT2**	**MT3**
100	0.47	0.48	0.57
200	1.15	1.16	1.26
300	2.21	2.20	2.30
400	3.65	3.63	3.74
500	5.50	5.47	5.61
600	7.83	7.76	6.53
700	10.78	10.56	10.76
800	14.33	14.10	14.34
900	18.70	18.28	18.64
1000	24.01	23.33	23.84

**Table 5:** Total inverter losses, $R_{TH}$ = 8.4 °C/W, thermal steady-state condition, $N_{S}/N_{P}$ = 9.5 (upper) and $N_{S}/N_{P}$ = 11.9 (lower).

$F_{\mathrm{OUT}}$ = 30 kHz			
**Power (W)**	**MT1**	**MT2**	**MT3**
800	14.33	14.10	14.34
900	18.70	18.28	18.64
1000	24.01	23.33	23.84
**800**	**22.29**	**21.98**	**22.1**
**900**	**29.27**	**28.69**	**29**
**1000**	**37.64**	**36.81**	**37.2**

## Data Availability

Not applicable.
